# Breathomics in Asthmatic Children Treated with Inhaled Corticosteroids

**DOI:** 10.3390/metabo10100390

**Published:** 2020-09-29

**Authors:** Valentina Agnese Ferraro, Silvia Carraro, Paola Pirillo, Antonina Gucciardi, Gabriele Poloniato, Matteo Stocchero, Giuseppe Giordano, Stefania Zanconato, Eugenio Baraldi

**Affiliations:** 1 Department of Women’s and Children’s Health, University of Padova, 35128 Padova, Italy; silvia.carraro@unipd.it (S.C.); paola.pirillo@gmail.com (P.P.); Antonina.gucciardi@unipd.it (A.G.); gabriele.poloniato@studenti.unipd.it (G.P.); matteo.stocchero@unipd.it (M.S.); giuseppe.giordano@unipd.it (G.G.); stefania.zanconato@aopd.veneto.it (S.Z.); eugenio.baraldi@unipd.it (E.B.); 2 Institute of Pediatric Research (IRP), Fondazione Città della Speranza, 35128 Padova, Italy

**Keywords:** pediatric asthma, breathomics, inhaled corticosteroids, endogenous steroid profile

## Abstract

Background: “breathomics” enables indirect analysis of metabolic patterns underlying a respiratory disease. In this study, we analyze exhaled breath condensate (EBC) in asthmatic children before (T0) and after (T1) a three-week course of inhaled beclomethasone dipropionate (BDP). Methods: we recruited steroid-naive asthmatic children for whom inhaled steroids were indicated and healthy children, evaluating asthma control, spirometry and EBC (in asthmatics at T0 and T1). A liquid-chromatography–mass-spectrometry untargeted analysis was applied to EBC and a mass spectrometry-based target analysis to urine samples. Results: metabolomic analysis discriminated asthmatic (*n* = 26) from healthy children (*n* = 16) at T0 and T1, discovering 108 and 65 features relevant for the discrimination, respectively. Searching metabolomics databases, seven putative biomarkers with a plausible role in asthma biochemical–metabolic processes were found. After BDP treatment, asthmatic children, in the face of an improved asthma control (*p* < 0.001) and lung function (*p* = 0.01), showed neither changes in EBC metabolomic profile nor in urinary endogenous steroid profile. Conclusions: “breathomics” can discriminate asthmatic from healthy children, with prostaglandin, fatty acid and glycerophospholipid as putative markers. The three-week course of BDP—in spite of a significant clinical improvement—was not associated with changes in EBC metabolic arrangement and urinary steroid profile.

## 1. Introduction

Pediatric asthma is a heterogeneous chronic inflammatory disease of the airways, characterized by a large number of observable characteristics (phenotypes) related to a complex combination of underlying pathophysiological and/or molecular mechanisms (endotypes), which involve several cell types, mediators and immune pathways [1,2,3]. Endotypes differ across the spectrum of asthma, providing a variable response to standard anti-inflammatory therapy with inhaled corticosteroids (ICS) [4,5], which are presently the mainstream of asthma treatment [6,7,8]. Furthermore, different regulatory mechanisms, such as release of a variety of cytokines, chemokines and other mediators, may influence airway epithelium responses [9]. The different endotypes have been investigated in recent years also through highly sophisticated analysis techniques, such as the “-omic sciences”, which have the potential to identify markers useful either in the diagnosis or as a guide for treatment [10]. As one of these“-omic sciences”, metabolomics enables the identification of the metabolomic profile associated with a specific condition by comparing different group of subjects [10,11,12,13].

A number of studies have applied the metabolomic approach in the analysis of different biofluids to characterize asthmatic subjects [10,14,15,16,17]. An interesting application is the metabolomic analysis of exhaled breath condensate (“breathomics”)—a biologic fluid obtained by cooling down exhaled air with a composition that mirrors the physio-pathologic processes of the lung [18,19]. Exhaled breath condensate (EBC) analysis enables an indirect noninvasive assessment of the lung and it is a technique with promising application, especially in pediatrics [19]. In fact, for pediatricians, it is very important to develop diagnostic techniques not requiring a high level of cooperation since even the application of spirometry, a key diagnostic technique in asthma, can be limited by poor children’s cooperation.

The main aim of the present study was to analyze through a metabolomic approach the EBC collected in asthmatic children, having a group of healthy children as control. Asthmatic children were evaluated before (at recruitment, T0) and after a three-week course (T1) of inhaled beclomethasone dipropionate (BDP). To our knowledge no previous studies investigated the effects of pharmacotherapy on metabolic fingerprints in pediatric asthma.

Secondary aim was to compare the levels of endogenous urinary steroids in recruited asthmatic and healthy children.

## 2. Results

### 2.1. Study Population

Twenty-six asthmatic children were recruited (median age 9.1 years, IQR 6.5–13.4), of whom 20 (76.9%) were male. Twenty-three patients were sensitized to at least one airborne allergen, as demonstrated by skin prick tests or specific serum IgE levels.

16 healthy children were recruited as controls (median age 10.2, IQR 6.2–14.4), of whom 11 (68.8%) were male.

Patients characteristics are shown in Table 1.

### 2.2. Symptoms Control and Lung Function

At baseline, asthma was defined as uncontrolled in 13 (50%) asthmatic children and partly controlled in the remaining 13 (50%) asthmatic children. After three weeks of treatment with inhaled BPD 100 mcg b.i.d. a significant improvement in the level of asthma control was detected (*p* < 0.001). In particular, asthma was well controlled in 20 (76.9%) children, four children improved from uncontrolled to partly controlled asthma, two remained partly controlled.

As far as it concerns lung function, FEV1 was significantly higher (*p* = 0.01) after the course of BDP than at recruitment, while no significant change was found in the remaining spirometric parameters (α = 0.05 with Bonferroni correction) (Table 1).

### 2.3. Metabolomics Analysis

A data set composed of 108 and a data set of 169 time@mass variables were obtained applying the negative and positive-ionization mode, respectively. No outliers were detected on the basis of the PCA model of each group considering a significance level α = 0.05 for the T2 test and the Q test.

#### 2.3.1. Asthmatic Group at Recruitment (T0) vs. Controls

Univariate data analysis pointed out 108 variables (53 in negative and 55 in positive-ionization mode) as being significantly different between the two groups.

**Figure 1:**
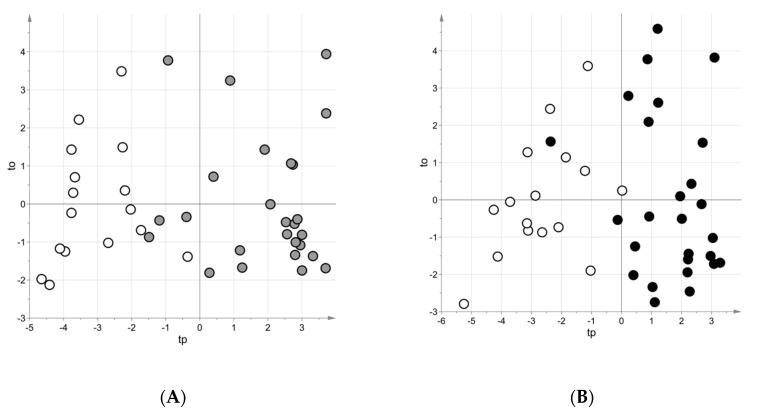
Score scatter plots of the projection to latent structures-regression-discriminant analysis (PLS-DA) models after post-transformation obtained for the data in negative-ionization mode. (**A**) Group T0 vs. controls (108 variables, A = 2 latent variables, MCC in 5-fold cross-validation = 0.76); (**B**) Group T1 vs. controls (108 variables, A = 2 latent variables, MCC in 5-fold cross-validation = 0.65). White circles—controls; gray circle—samples of Group T0; black circles—samples of Group T1 as.

Since PLS–DA (projection to latent structures regression–discriminant analysis) models were not affected by structured noise, variable influence on projection (VIP) was used as ranking parameter in the stability selection procedure [20]. Considering 200 random subsamples extracted by bootstrap, the investigation of the negative-ionization dataset led to a Matthews correlation coefficient (MCC) for the out-of-bag predictions (MCCoob) equal to 0.72 and pointed out 14 discriminating variables, whereas the analysis of the positive-ionization dataset highlighted 10 discriminating variables with a MCCoob equal to 0.59. The score scatter plot of the PLS-DA model obtained considering the negative-ionization dataset is reported in Figure 1A (a similar plot was obtained for the positive-ionization dataset, data not shown). All the variables highlighted by multivariate data analysis were pointed out by univariate data analysis.

#### 2.3.2. Asthmatic Group after Three Weeks of Treatment (T1) vs. Controls

**Figure 2:**
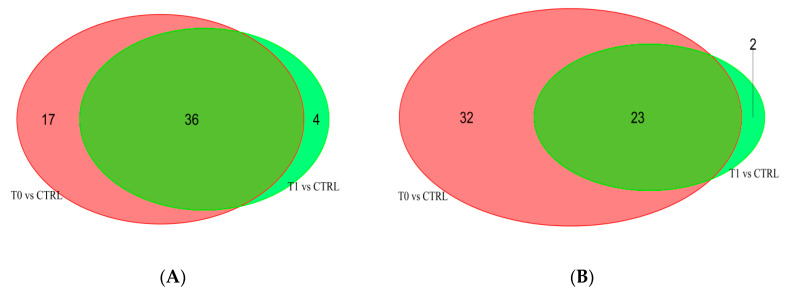
Share and unique relevant variables. (**A**) Results for the dataset obtained by negative-ionization mode; (**B**) results for the dataset from positive-ionization mode.

Univariate analysis enables the identification of 65 variables (40 detected in negative and 25 in positive-ionization mode) significantly different between the two groups. Thirty-six variables of the 40 detected in negative-ionization mode and 23 variables of the 25 in positive-ionization mode were the same highlighted comparing asthmatic group at recruitment vs. controls, as reported in Figure 2.

Through the multivariate approach (considering 200 random subsamples and VIP as importance measure because structured noise was not detected in the models), analyzing the negative-ionization dataset 14 discriminating variable were found with a MCCoob equal to 0.53, whereas analyzing the positive-ionization dataset nine relevant variables were detected and MCCoob was equal to 0.31. The score scatter plot of the PLS-DA model obtained considering the data in negative-ionization mode is reported in Figure 1B (a similar plot was obtained for the positive-ionization dataset, data not shown). All the variables highlighted by multivariate data analysis were pointed out by univariate data analysis.

#### 2.3.3. Asthmatic Group at Recruitment (T0) vs. Asthmatic Group after Three Weeks of Treatment (T1)

To compare the metabolomic profile in asthmatic children at recruitment and after a course of BDP, paired methods have been applied. The univariate analysis found no variable significantly different between T0 and T1. Likewise, the multivariate analysis found no models with a reliable performance in prediction (MCC in 5-fold cross-validation and MCCoob less than 0.1).

#### 2.3.4. Putative Markers Annotation

The main available metabolomic databases (Human metabolome database (HMDB) and METLIN) were searched to annotate the relevant variables in order to find a plausible biologic significance characterizing each group.

Searching the HMDB for the relevant discriminant variables, a set of 69 variables were putatively annotated. Among them, six were associated with metabolites possibly associated with biochemical–metabolic processes that characterize asthma (Table 2). All these variables resulted lower in controls than in the other two groups (T0 and T1).

#### 2.3.5. Target Metabolomics Analysis: Urinary Steroids

Through a target mass spectrometry approach the endogenous products of steroid metabolism were evaluated. The following metabolites were quantified in positive-ionization mode: progesterone, androstenedione, 20β-dihydrocortisone, androsterone, dehydroepiandrosterone, estrone, 21-hydroxyprogesterone, 17-hydroxyprogesterone, testosterone, β-estradiol, 11-deoxycortisol, corticosterone, cortisone, cortisol, estriol, 7α-hydroxydehydroepiandrosterone, 11-ketoetiocholanolone, 11-ketoandrostenedione, 11β-hydroxyandrostenedione, 20β-dihydrocortisone. In negative-ionization mode, the following metabolites were quantified: etiocholanolone glucuronide, dehydroepiandrosterone glucuronide, tetrahydrocortisone, allotetrahydrocortisol, 5β-dihydrocortisol, α-cortolone, β-cortolone, β-cortol, dehydroepiandrosterone sulfate, epitestosterone sulfate, epiandrosterone sulfate, etiocholanolone sulfate, androsterone sulfate.

Applying the same data analysis procedure discussed for the untargeted metabolomics investigation, no differences in the steroid arrangement were discovered between the three groups under investigation.

In urinary samples collected in asthmatic children after the course of inhaled steroid therapy, BDP and its metabolites were not detected.

## 3. Discussion

This study applied metabolomic analysis to EBC (“breathomics”) in asthmatic children (before and after a course of inhaled steroid treatment) and in a control group of healthy children. On the basis of the metabolomic profile asthmatic children could be discriminated from healthy controls both at baseline and after a three-week course of BDP treatment. On the other hand, in spite of a significant clinical and functional improvement, no significant changes in the metabolomic arrangement were found in asthmatic children after the course of BDP. Interestingly, no BDP or its metabolites were found in the urine of asthmatic children and patients’ endogenous steroid metabolism seems not to be affected by the treatment.

Understanding underlying processes of pediatric asthma through the “-omic” sciences is one of the challenges of the years to come. In keeping with previous studies [19,20,21,22], we demonstrated that “breathomics” enables a clear discrimination between steroid-naïve asthmatic children and healthy controls, as shown by the robust PLS-DA model found. Moreover, we described a robust PLS-DA model for discriminating between asthmatic patients after three weeks of BDP and healthy controls, showing that, even if regularly treated with inhaled steroids, asthmatic children still have a lung metabolic profile different from healthy controls.

Searching the available databases, we found some metabolites, increased in asthmatic subjects, with a plausible biologic role in asthma biochemical-metabolic processes.

Two metabolites belong to prostaglandins, one (*m*/*z* 415.255) being increased both at T0 and at T1, while the other (*m*/*z* 295.154) only at T0. Interestingly, from a metabolomic standpoint, the present study confirms a previous one from our group [23], in which the role of prostaglandins already emerged in the characterization of a group of children with non-severe asthma. This result confirms that metabolomic approach applied to EBC has the potential to identify metabolites actually relevant in asthma biopathology. In fact, the role of prostaglandins in asthma is well recognized [24]: a higher concentration of prostaglandin D2 (PGD2) was described in the bronchoalveolar (BAL) fluid of allergic asthmatic patients acting as a potent bronchoconstrictor, vasodilator and potentiating airway responsiveness [24]; prostaglandin E2 (PGE2) is known as one of the most plentiful COX products in airway epithelium and smooth muscle, acting as an inhibitor of vagal cholinergic contraction of airway smooth muscle and suppressor of proinflammatory cytokine expression [25,26]; an abundant production of prostaglandin I2 (PGI2) was outlined in allergic inflammatory responses in the lung restraining airway inflammation [24].

In addition, our data show persistently increased levels of at least one of these metabolites even after the course of BDP, supporting the poor effect of steroids on the release of prostaglandin, as previously showed in vitro after the incubation of lung parenchyma with steroids [27].

Some metabolites belonging to fatty acids metabolism were also identified in the EBC of asthmatic children. Higher levels of *N*-acyl amides (*m*/*z* 371.228) and omega-amino fatty acids (*m*/*z* 198.186) were detected both before and after BDP compared to healthy controls and higher level of omega-amino fatty acids (*m*/*z* 172.133) was found at T0 compared to healthy controls. These results confirm that fatty acids can be involved in asthma pathogenetic mechanisms, as suggested by a previous study that applied a liquid chromatography-high-resolution mass spectrometry-based metabolomic approach to analyze serum samples [28]. Fatty acids in asthma can affect the equilibrium between pro- and anti-inflammatory mechanisms: n-3 polyunsaturated fatty acids (PUFAs) or monounsaturated fatty acids (MUFAs) could reduce inflammation, while saturated fatty acids (SFAs) may contribute to proinflammatory responses [29]. PUFAs seem also to be associated with a lower risk of allergic disease development in early childhood years, playing a protective role against allergy development [30,31]. Furthermore, as suggested by our data, also a previous study did not show correlations between fatty acid profile and ICS treatment [28]. The persistence of metabolic abnormalities despite steroid treatment suggests the role of steroid-insensitive mechanisms in the biochemical–metabolic processes that characterize asthma.

Finally, higher levels of mono glycerophospholipids (*m*/*z* 520.334) were detected in EBC of asthmatic children after BDP than in healthy children. Glycerophospholipid metabolism has been shown to be altered in BAL, lung tissue and serum from mouse model of allergic asthma [32,33] and in BAL from asthmatic subjects and mice [34,35], pointing out the possible role of glycerophospholipid in the pathogenesis of asthma.

In spite of a significant clinical and functional improvement, we found no significant difference in the metabolomic profile within the group of asthmatic children after BDP. This result is in line with a previous study of our group, where a cross-sectional analysis could not discriminate children with mild asthma regularly treated with inhaled steroids from those steroid naïve [23]. This may suggest that low dose inhaled steroid therapy, although clinically effective, does not modify significantly the metabolic-inflammatory processes underlying asthma. It is plausible, instead, that inhaled therapies used at higher doses and/or for a longer period could significantly modify the metabolic profile, as already demonstrated for gene expression profile [36]. Another possible explanation is that changes induced by the treatment are limited to specific metabolic pathways or too subtle to be detected through a metabolomic approach. However, the investigation of EBC metabolomic profiles has the potential to help the characterization of the mechanisms that underlie asthma and may drive a precision therapy approach [37].

As for the secondary objective of the study, interestingly the target analysis conducted on urine samples demonstrated that the three-week course of inhaled BDP does not have significant effect on steroid endogenous metabolism. Moreover, no BDP or derived metabolites could be found in urine above our limit of detection. These results support the safety of low doses of BDP described in previous studies [38,39,40].

Potential limitations of our study mainly concern the small sample size and the short period of inhaled steroid treatment applied. About the sample size, due to the lack of preliminary information about the effect size, we were not able to perform power analysis to estimate the right sample size to use and, then, we recruited groups composed of 15–20 subjects according to the common practice applied to metabolomics pilot studies. We acknowledge that this approach may introduce bias in the results. On the other hand, our preliminary results could be used to design further studies where sample size is correctly estimated and other therapeutic strategies (doses higher or given for longer periods) are applied. These new investigations are necessary to validate our preliminary results. Moreover, the role of the putative biomarkers identified needs to be confirmed in studies applying also targeted approaches.

Another potential limit of the study is the lack of a control group of asthmatic children receiving placebo. Although the inclusion of such group would have improved the study design, it would be unethical giving placebo to symptomatic asthmatic children in whom ICS therapy is indicated.

## 4. Materials and Methods

### 4.1. Study Population

Asthmatic children, 5 to 14 years of age, were enrolled prospectively and consecutively between January 2018 and January 2019 from among the outpatients attending the Unit of Pediatric Allergy and Respiratory Medicine, Department of Women’s and Children’s Health, Padova. Children with steroid naïve asthma who had to start a Step 2 therapy with low dose inhaled steroids to control symptoms and minimize future risk, according to Global Initiative for Asthma 2018 [41], were included. Thus, asthmatic children were included if they had a characteristic pattern of respiratory symptoms (such as wheezing, shortness of breath, chest tightness or cough), requiring the start of an ICS-based therapy [41]. Exclusion criteria included preterm birth or personal history of respiratory distress at birth or chronic diseases other than asthma or use of any steroid treatment in the previous month prior to enrolment.

We also enrolled a group of healthy children (6–15 years of age), with no history of allergic or respiratory diseases.

The study was approved by the Ethics Committee of Padova General Hospital (Protocol no. 57776), and all parents gave their written informed consent to their children’s participation in the study.

### 4.2. Study Design

The study implements a case-control design. At recruitment (T0), all the children (both asthmatic and control subjects) underwent anamnestic investigation, physical examination, spirometry, EBC and urine sample collection. At recruitment asthmatic children were prescribed beclometasone dipropionate (HFA) 100 mcg b.i.d. via a spacer device and mouthpiece. After 3 weeks (T1) they were revaluated and underwent spirometry, EBC and urine sample collection.

At each evaluation, according to GINA guidelines [41], asthma was classified as well controlled, partially controlled or uncontrolled depending on the presence of daytime symptoms, night wakening, need for relievers and limitation to physical activity.

Lung function was measured with a 10-L bell spirometer (Biomedin, Padua, Italy). At least three spirometric maneuvers were completed, with at least two reproducible maneuvers required for each test. The most accurate FVC and FEV1 of the three maneuvers were considered for data analysis. All spirometric values were analyzed using Z-score according to reference values of the Global Lung Function Initiative powered by European Respiratory Society [42,43].

### 4.3. Metabolomic Analysis

#### 4.3.1. EBC and Urine Collection

For all recruited children, EBC was collected and processed according to European Respiratory Society recommendations [44], using a modified TURBO-DECCS (a transportable unit for use in research on markers obtained from disposable exhaled condensate collection systems; Medivac). An EBC sample volume of 1.5 mL was collected in 15 min of tidal breathing. To avoid any contamination of EBC from undefined environmental conditions, we adapted a filter (Honeywell Filters, Honeywell Respiratory Safety Products, Paris, France) on the inspiratory valve, as recently recommended [44]. After the visit, EBC samples were immediately stored at −80 °C for subsequent analysis.

A urine sample was collected from all children, using a sterile urine cup, and urine samples were stored at −80 °C until metabolomic analysis.

#### 4.3.2. UPLC-MS (Ultra-Performance Liquid Chromatography-Mass Spectrometer) Analysis of EBC

EBC samples were analyzed at the Laboratory of Mass Spectrometry and Metabolomics of the Department of Women’s and Children’s Health, University of Padova. The metabolic profile of the EBC was acquired using a Waters Acquity ultra-performance liquid chromatography (UPLC) system coupled to a Waters Q-TOF Synapt G2 mass spectrometer (Waters, Milford, MA, USA) with an electrospray source in both positive and negative-ionization mode.

A full description of the analytical procedure is provided in Appendix A (Table A1 and Table A2).

#### 4.3.3. Analysis of Urinary Steroids and BDP

Steroids were extracted from 250 µL of urine added of labeled internal standards (MassChrom^®^ Steroids, ChromSystems, Gräfelfing, Germany) by solid phase extraction (Oasis HLB cartridges, 30 mg, 1 mL) and reconstituted in 100 μL of methanol. Mass spectrometric analyses were performed using a Xevo TQ-S tandem quadrupole mass spectrometer (MS/MS) (Waters Co., Milford, MA, USA) equipped with an electrospray ion source coupled with an UPLC Acquity Waters (Manchester, England).

A full description of the analytical procedure is provided in Appendix B.

#### 4.3.4. Data Preprocessing

Untargeted metabolomics raw data were extracted by Progenesis software (Waters Corporation, Milford, MA, USA). All the extracted time@mass features showing a coefficient of variation greater than 25% in the quality-control samples were excluded. Missing data were imputed generating a random number between zero and the minimum value recorded for the feature. Probabilistic quotient normalization was applied. Data were log-transformed and mean centered.

For target metabolomics, in the case of determination below the limit of quantification, a random number between zero and the limit of quantification was generated. Data were auto scaled.

#### 4.3.5. Statistical Data Analysis

Three groups of subjects were defined: the group of asthmatic children at recruitment (T0), the group of asthmatic children after 3 weeks of treatment (T1) and the control group.

The population and the spirometric data were investigated applying t-test or paired t-test depending on the compared groups and Fisher’s exact test for qualitative features. In the case of multiple comparison, Bonferroni correction was applied.

For the metabolomics investigation, data were analyzed by pair-wise comparison applying univariate and multivariate data analysis. Specifically, the control group was compared to asthmatic group at T0 and at T1 to discover modifications in the metabolic composition of EBC using unpaired statistical methods. Univariate data analysis was based on Mann–Whitney test controlling the false discovery rate (FDR) by the Benjamini-Hochberg procedure at level δ = 0.10 [45]. Multivariate data analysis was based on projection methods. Principal component analysis (PCA) was applied for exploratory data analysis and to detect outliers (T2 test and Q test, α = 0.05) whereas the differences in the metabolic profiles of EBC were investigated by projection to latent structures–regression-discriminant analysis (PLS-DA) with stability selection [20]. Relevant features were identified assuming a significance level α = 0.05. Five-fold cross-validation and permutation test on the class (1000 random permutations) were applied to check over-fitting. PLS-DA models were post-transformed to simplify model interpretation [46].

Moreover, methods of paired comparison were applied to compare asthmatic children evaluated at T0 and at T1. Specifically, Wilcoxon test with FDR (Benjamini-Hochberg procedure at level δ = 0.10) was applied, whereas multivariate data analysis was based on multilevel-PLS-DA with stability selection [47].

Data preprocessing and data analysis were performed using in-house R-functions implemented by the R 3.6.0 platform (R Foundation for Statistical Computing, Vienna, Austria).

The relevant variables selected by multivariate data analysis were merged with those obtained from the univariate data analysis. Thus, relevant variables were identified by searching the Human Metabolome Database and the METLIN metabolite database.

## 5. Conclusions

In the present study, a “breathomic” approach could clearly discriminate asthmatic from healthy children.

Within the discriminating profile metabolites belonging to prostaglandin, fatty acid and glycerophospholipid metabolism were identified as putative markers, with a potential role in biochemical-metabolic processes that characterize asthma.

The three-week course of BDP—in spite of leading to a significant clinical improvement—was not associated with significant modification in the EBC metabolic arrangement.

Further studies are needed to evaluate whether other therapeutic approaches (longer treatment or higher steroid doses) could have a significant metabolomic effect.

## Figures and Tables

**Table 1:** Characteristics and spirometric values (expressed as percent of predicted values) of the recruited subjects.

Heading		Asthmatic Children		Healthy Children	*p* Value
Age (year)		9.1 (6.5, 13.4)		10.2 (6.2,14.4)	0.16
Sex (male/female)		20/6		11/5	0.72
BMI		17.0 (14.2, 21.7)		17.9 (14.3, 25.0)	0.18
Allergic (yes/no)		23/3		0/16	<0.001
		T0	T1		
FEV1	Z-score % predicted	−0.59 (−2.54, +1.38) 92.87 (69.19, 116.55)	−0.35 (−2.07, +1.37) 95.69 (75.73, 115.65)	−0.16 (−1.54, +1.22) 97.99 (81.67, 114.31)	0.07 ^a^ 0.23 ^b^ 0.01 ^c^
FEF 25–75	Z-score % predicted	−0.22 (−2.06, +1.62) 95.71 (52.27, 139.15)	−0.10 (−2.08, +1.88) 98.52 (50.76, 146.28)	0.33 (1.23, 1.89) 108.49 (70.75, 146.23)	0.02 ^a^ 0.07 ^b^ 0.08 ^c^

Data shown as median and (10th, 90th) percentile; ^a^—at recruitment (T0) versus healthy; ^b^—after treatment (T1) versus healthy; ^c^—T0 versus T1. FEV1 = forced expiratory volume in 1 s; FVC = forced vital capacity.

**Table 2:** Annotation of the features involved in the discrimination between asthmatic and healthy children.

*m*/*z*	Retention Time (min)	Adduct	Annotation	Class
172.1332	4.42	M − H	9-amino-nonanoic acid	omega-amino fatty acids
198.1864	4.92	M + H − H_2_O	12-amino-dodecanoic acid	omega-amino fatty acids
295.1543	5.53	M − H	lactone of PGF-MUM	prostaglandins
371.2280	2.84	M + H − NH_3_, M + H	*N*-linoleoyl taurine	*N*-acyl amides
415.2546	3.12	M + H − NH_3_, M + H	17-phenoxy trinor PGF2α ethyl amide	prostaglandins
520.3342	3.58	M + H − NH_3_, M + H	lysoPC (18:2(9*Z*,12*Z*))	mono glycerophospholipids

All features annotated as Level 3. PGF-MUM = delta-lactone of 5 alpha-7 alpha-dihydroxyketotetranorprosta-1,16-dioic acid; lysoPC = Lysophosphatidylcholine.

## Appendix A

### UPLC-MS Analysis of EBC

EBC samples were analyzed at the Laboratory of Mass Spectrometry and Metabolomics of the Department of Women’s and Children’s Health, University of Padova.

After being thawed at room temperature, 450 µL of EBC samples, were dried at 30 °C by a rotary evaporator (Labconco, Kansas City, MO, USA) and added of 150 µL of water and 0.1% of formic acid (H_2_O + 0.1% FA). The same procedure was applied to quality-control samples (QC), obtained mixing together 20 µL of each sample.

QC and standard mix solution were used to test for reproducibility and accuracy during the analysis. Blank samples were H_2_O + 0.1% FA. Samples, QCs, standard mix solutions and blanks were randomly injected to prevent any spurious classification.

The metabolic profile of the EBC was acquired using a Waters Acquity ultra-performance liquid chromatography (UPLC) system coupled to aWaters Q-TOF Synapt G2 mass spectrometer (Waters, Milford, MA, USA) with an electrospray source (ESI) in both positive (ESI+) and negative (ESI−)^−^ ionization mode. The sample enrichment was made by a coupled Rheodyne valve with a C18 cartridge (2.1 × 30 mm, 1.7 μm, Waters, Milford, MA, USA) to enhance the sensitivity of compounds detection. The chromatographic separation was performed using a C18 column (1 × 100 mm, 1.7 µm, Waters, Milford, MA, USA).

The solvents used for elution in C18 column were H_2_O + 0.1% FA (Phase A) and acetonitrile:methanol 80:20 + 0.1% FA (Phase B). For the enrichment C18 cartridge, H_2_O + 0.1% FA (Phase A) was used for activation and acetonitrile (Phase B) for washing the cartridge. The C18 column and the cartridge were maintained at 65 °C with a flow of 0.2 mL/min. Twenty microliters of each sample were injected by the autosampler kept at 6 °C. The chromatographic gradients were reported in Table A1 and Table A2.

For the detection of the ions in the Q-TOF, the mass scan was set in a range between 20 and 1200 *m*/*z* and data were collected in continuum mode. For mass accuracy, a LockSpray interface was used to perform mass correction.

**Table A1:** Gradient of the mobile phase in the C18 column.

Time (min)	Flow (mL/min)	% A	% B
initial	0.2	90	10
1.00	0.2	90	10
3.50	0.2	70	30
6.50	0.2	5	95
8.00	0.2	5	95
11.00	0.2	90	10

**Table A2:** Activation gradient for C18 cartridge.

Time (min)	Flow mL/min	% A	% B
Initial	0.500	98	2
0.50	0.500	98	2
1.50	0.500	0.0	100
2.00	0.500	0.0	100
3.00	0.500	0.0	100
5.00	0.500	98	2
10.00	0.200	98	2
10.01	0.200	98	2
11.00	0.500	98	2

## Appendix B

### Analysis of Urinary Steroids and BDP

Steroids were extracted from 250 µL of urine added of labeled internal standards (MassChrom^®^ Steroids, ChromSystems, Gräfelfing, Germany) by solid phase extraction (Oasis HLB cartridges, 30 mg, 1 mL) and reconstituted in 100 μL of methanol. Mass spectrometric analyses were performed using a Xevo TQ-S tandem quadrupole mass spectrometer (MS/MS) (Waters Co., Milford, MA, USA) equipped with an electrospray ion source operating in positive and negative-ionization mode, coupled with an UPLC Acquity Waters (Manchester, England).

The chromatographic separation was performed on a UPLC HSS T3 (1.7 μm, 2.1 × 100 mm) fitted with a VanGuard™ 2.1 × 5 mm pre-column, by solutions of H_2_O + 0.1% FA and methanol:acetonitrile 90:10 vv^−1^ with 0.1% FA. Two different gradients were used for compounds elution for the positive and negative analysis. Multiple reaction monitoring (MRM) acquisition mode was selected for the quantification of the 53 steroids examined in this study. Data processing was done using TargetLynx software and metabolites were quantified using area ratio (i.e., area of metabolite/area of internal standard) and external calibration curves. Urinary steroid concentrations were normalized to creatinine estimated by Jaffe’s alkaline picric method.

The analysis of urine samples for BDP, beclomethasone 17-monopropionate (17-BMP) and beclomethasone (BOH) was performed by UPLC-MS/MS. We used the same extraction and chromatographic conditions described above for steroids analysis to assay BDP and its metabolites. Protonated molecules were used as precursor ions with selected reaction monitoring of the following transitions *m*/*z* 521→319, *m*/*z* 465→279 and *m*/*z* 409→279 for BDP, 17-BMP and BOH, respectively. The limit of quantification for each analyte was 0.1 nM.
